# Apolipoprotein E-Mimetics Inhibit Neurodegeneration and Restore Cognitive Functions in a Transgenic Drosophila Model of Alzheimer's Disease

**DOI:** 10.1371/journal.pone.0008191

**Published:** 2009-12-07

**Authors:** Svetlana Sarantseva, Svetlana Timoshenko, Olga Bolshakova, Eugenia Karaseva, Dmitry Rodin, Alexander L. Schwarzman, Michael P. Vitek

**Affiliations:** 1 Petersburg Institute of Nuclear Physics, Russian Academy of Sciences, Gatchina, Russia; 2 Institute for Experimental Medicine, Russian Academy of Medical Sciences, St. Petersburg, Russia; 3 Division of Neurology, Duke University Medical Center, Durham, North Carolina, United States of America; 4 Cognosci, Inc., Research Triangle Park, North Carolina, United States of America; Massachusetts General Hospital and Harvard Medical School, United States of America

## Abstract

**Background:**

Mutations of the amyloid precursor protein gene (*APP)* are found in familial forms of Alzheimer's disease (AD) and some lead to the elevated production of amyloid-β-protein (Aβ). While Aβ has been implicated in the causation of AD, the exact role played by Aβ and its APP precursor are still unclear.

**Principal Findings:**

In our study, *Drosophila melanogaster* transgenics were established as a model to analyze AD-like pathology caused by *APP* overexpression. We demonstrated that age related changes in the levels and pattern of synaptic proteins accompanied progressive neurodegeneration and impairment of cognitive functions in APP transgenic flies, but that these changes may be independent from the generation of Aβ. Using novel peptide mimetics of Apolipoprotein-E, COG112 or COG133 proved to be neuroprotective and significantly improved the learning and memory of APP transgenic flies.

**Conclusions:**

The development of neurodegeneration and cognitive deficits was corrected by injections of COG112 or COG133, novel mimetics of apolipoprotein-E (apoE) with neuroprotective activities.

## Introduction

Alzheimer's disease (AD) represents the most common neurodegenerative disease occurring in mid-to-late life [1]. Clinically, AD manifests as a gradual decline of cognitive functions such as learning and memory, which significantly correlates with synaptic loss [2]–[4]. The mechanism(s) by which synaptic dysfunction occurs is not well understood. However, numerous results show that synaptic dysfunction occurs in the very early stages of many neurodegenerative diseases and precedes accumulation of abberant protein aggregates [5]–[8]. Furthermore, studies with transgenic animals recapitulated very similar early pathologic events for different disease models [9]–[12].

Animal models of AD typically mimic the pathogenesis of early onset familial AD that are caused by mutations in the amyloid precursor protein (*APP*) and presenilin (*PS1* and *PS2*) genes [13]. APP is a large, type I transmembrane protein, which is cleaved by different membrane associated proteases known as α, β and γ-secretases [14]. The coordinated action of β– and γ–secretases result in the formation of a 40–42 amino acid peptide named as amyloid beta protein (Aβ) whose beta pleated sheet conformation is a main constituent of amyloid plaques in AD brain [15]. In contrast, α -secretase cleaves APP in the middle of the Aβ region and prevents its production [14]. Additionally, it has been shown that PS1 is an integral part of the γ-secretase protease consisting of at least four different proteins in a membrane associated complex [16].

In most animal models of AD, overexpression of APP, with or without mutated presenilin expression, yields elevated levels of Aβ production and accumulation of oligomeric Aβ species that may contribute to synaptic failure and cognitive deficits [4].

Although APP overexpression and Aβ deposition in transgenic animals do not faithfully recapitulate all aspects of AD, they offer real opportunities for studying AD related neuropathology. In particular, these animal models help to explain the contributions of APP and Aβ in familial AD pathogenesis. In this respect, transgenic *Drosophila* models have several advantages for investigation of AD pathology. First, the *Drosophila* APP homologue, an APP-like protein (APPL) lacks the Aβ peptide region and its processing does not lead to neurotoxic effects [17]. Second, because β-secretase (BACE) activity is not present in *Drosophila* [18]–[20], APP expression in transgenic flies allows one to discriminate between abnormal effects of exogenous human APP and secreted Aβ. More importantly, transgenic *Drosophila* can be used to better understand targets for protective treatments to preserve cognitive and functional abilities in AD patients.

We now report that overexpression of human *APP* in neural cells of *Drosophila* leads to synaptic dysfunction, neurodegeneration, Aβ secretion and Aβ aggregation in the fly brain. Novel neuroprotectors, known as mimetics of apolipoprotein E (apoE), block neurodegeneration and restore cognitive functions in transgenic *Drosophila*. Selection of apoE-mimetics derived from the receptor-binding region of apoE was based on the ability of these peptides to mimic the functional anti-inflammatory and neuroprotective effects of the intact apoE protein seen in different animal models of neurological diseases [21], [22]. Moreover, these peptides suppressed elevation of Aβ1-42 levels in mice after head injury [23]. In summary, apoE mimetics are useful tools to mark the functional connections between neurodegeneration, cognitive impairment, Aβ secretion and Aβ aggregation

## Results

### I. Modeling AD Neuropathology in *Drosophila*

#### APP expression and accumulation of Aβ in transgenic fly brain

**Figure 1:**
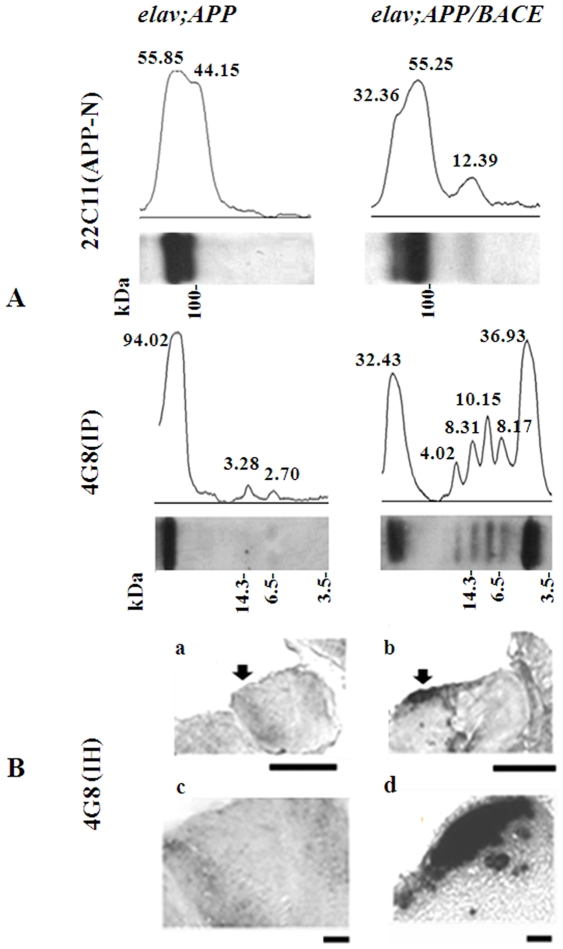
Human *APP* expression in the brain of transgenic *Drosophila*. **A**) Western blot analysis of APP and Aβ: 22C11 (APP-N): Full size human APP was detected by monoclonal antibody 22C11 (APP N terminal-specific); 4G8 (IP): Aβ was immunoprecipitated using anti-Aβ monoclonal 4G8 antibody; elav;APP; and -elav;APP/BACE- genotypes of transgenic strains used for analysis. All blots were scanned and relative intensity of bands was quantified using Image J software. **B**) Aβ deposits in *Drosophila* brain: 4G8 (IH): Immunohistochemistry with 4G8 antibody; Arrows indicate areas for magnification. Bar, 50 µm (a, b); Bar, 10 µm (c, d).

To express human *APP* genes in neuronal cells of *Drosophila*, we used the UAS/Gal 4 system [24]. In this system, the yeast transcriptional activator Gal 4 directs transcription of the transgene, which has been placed under the transcriptional control of an Upstream Activating Sequence (UAS). Flies carrying the *UAS-APP* transgene, in which a human *APP* cDNA was located downstream of the UAS promoter, were crossed to flies carrying a Gal 4 (*elav-GAL4^c155^*) transgene, which expresses predominantly in neurons [25]. Transgenes with the *elav;APP* genotype express APP at levels higher than the endogenous *APPL* gene [20], [26]. In *UAS-APP*-expressing lines, antibodies against the N-terminal region of human APP (22C11) detected full-length forms of APP695 [Fig. 1A-22C11(APP-N), *elav; APP*]. Double transgenic lines coexpressing human *APP* and *BACE* resulted in the partial loss of the larger APP band [Fig. 1A-22C11(APP-N), *elav; APP/BACE*]. Immunoprecipitation of SDS-protein extracts from the heads of *elav; APP/BACE* transgenics with monoclonal antibody 4G8 revealed Aβ monomer and SDS-stable Aβ oligomers [Fig. 1A-[Fig pone-0008191-g002] [Fig pone-0008191-g003] 4G8(IP), *elav APP/BACE*]. Aβ derived from human APP expression was detectable in immunoprecipitates at 2 days after eclosion. We did not detect Aβ monomer and Aβ oligomers in immunoprecipitates of flies with the *elav; APP* genotype, where staining was detected only at the top of gradient gel reflecting the position of IgG migration. Immunohistochemistry demonstrated that Aβ deposits in the *Drosophila* brain were detected mainly in the outer cellular cortex layer that contains neuronal and glial cells in flies simultaneously expressing *APP* and *BACE* [Fig. 1B-b,d; *elav; APP/BACE*].

#### Distribution of GFP–n-synaptobrevin in the transgenic *Drosophila* brain

Next, we studied distribution of n-synaptobrevin in brain sections of transgenic *Drosophila*. Synaptobrevin is one of the main presynaptic proteins and represents a reversible linker between synaptic vesicles and the cytoskeletal meshwork. Thus, the distribution of n-synaptobrevin in neural cells of *Drosophila* brain may demonstrate cytoskeletal integrity and illuminate possible abnormalities in synaptic functions. We used green fluorescent protein (GFP) fused in frame with n-synaptobrevin (n-syb-eGFP) as a detection reporter for its local distribution. Several transgenic lines were studied including flies expressing APP, APP-Swedish and N- or C–terminally truncated APPs. APP–*Swedish* is an APP695 transgene with the (670K3N, 671M3L) mutations found in familial AD [13]. The gene encoding APPΔ*CT* included sequences for the extracellular domain, Aβ, and the short membrane anchor KKKR, followed by a stop codon [27]. The APPΔ*NT* structure included the sequence of the APP signal peptide fused with an APP695 C-terminal fragment beginning from the residue N584 located at a distance of 12 amino acid residues upstream from the 5′-site of BACE cleavage [27]. Thus, Δ*NT* also included the Aβ sequence. The presence of the Aβ domain in all truncated APP forms studied here was necessary for axonal transport of the truncated APP to the presynaptic terminal [28].

**Figure 2 pone-0008191-g002:**
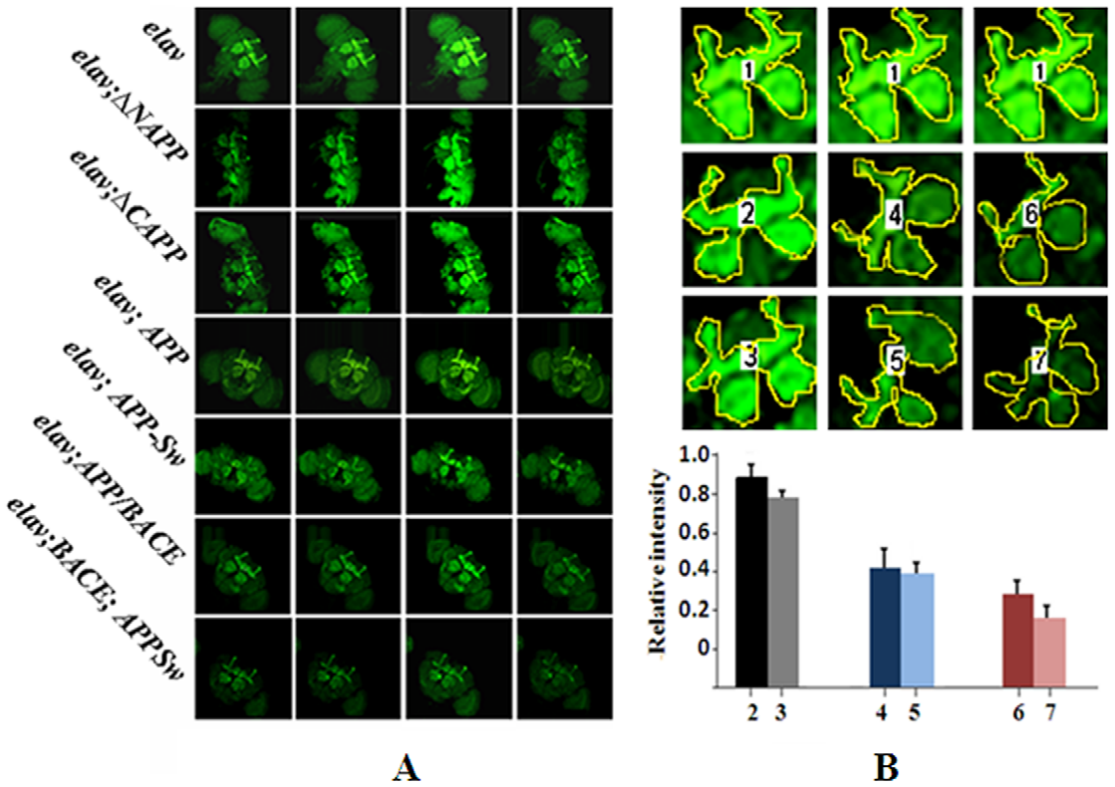
Distribution of GFP–n-synaptobrevin in the *Drosophila* brain. **A**) Optical sections of the brain obtained by confocal microscopy (four brightest sections for each genotype). The fluorescent signal was visualized at a wavelength of λ 488 nm. The scanning sections were 2 µm thick. Microphotographs show the brains of 30-day-old flies. **B**) Quantitation of relative intensities of GFP–n-synaptobrevin (n-syb-eGFP) in the brain section for transgenic flies with different genotypes: 1.-elav; 2- elav;ΔNAPP; 3- elav;ΔCAPP; 4-elav;APP; 5-elav;APPSw; 6-elav;APP/BACE; 7- elav; APPSw/BACE. The pixel intensity in the selected areas was estimated using Image J software and was normalized to the fluorescent signal of *elav* genotype. Relative intensities are shown as averages±SEM. p<0.05.

Laser confocal microscopy in transgenic lines expressing n-syb-eGFP showed that fluorescence is largely localized in the mushroom bodies, the brain region involved in associative learning and memory, and to the antennal lobe of the brain region, which is responsible for olfactory function [Fig. 2A]. A significant reduction in fluorescence intensity in mushroom bodies and antennal lobes was seen in transgenics expressing full size APPs and n-syb-eGFP in 30-day-old flies. The most significant reduction of fluorescence intensity in optical slices was detected in transgenics expressing *APP-Swedish* plus *BACE*. In contrast, reduction of n-syb-eGFP levels in flies expressing truncated APPs was not significant in 30-day old flies compared with those expressing n-syb-eGFP alone [Fig. 2A]. As an additional control, signal intensities of n-syb-eGFP in the mushroom bodies and in the antennal lobes were almost equal in all transgenic lines on the second day after hatching (data not shown). For calculation of relative intensities, all pixel intensities in the selected areas were normalized to the fluorescent signal/pixel intensities of the *elav* genotype. Relative intensities are shown as mean±SEM. Quantitation of n-syb-eGFP fluorescence demonstrates the following: first, transgenics expressing *APP* plus *BACE* had lower levels of presynaptic proteins than transgenics expressing only *APP* and second, overexpression of full length APP may be sufficient for abnormal synaptogenesis in the *Drosophila* brain.

#### Age-dependent neurodegeneration in transgenic *Drosophila*

**Figure 3 pone-0008191-g003:**
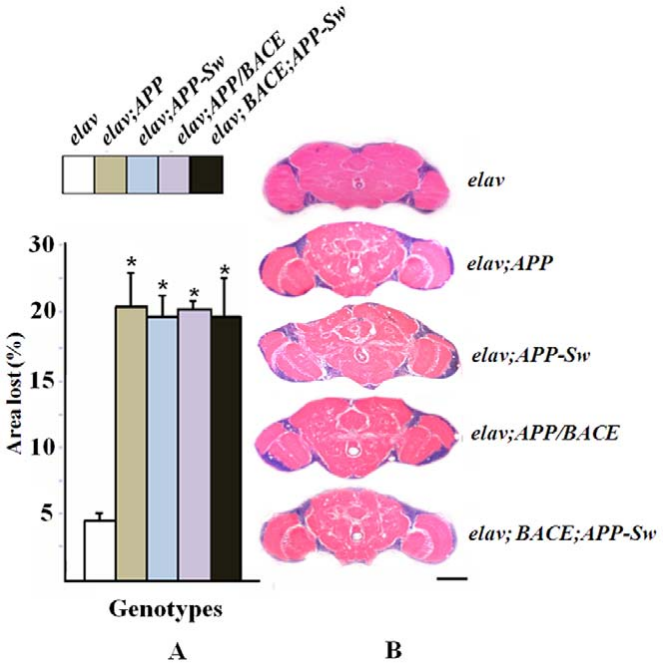
Age-dependent neurodegeneration in *APP* expressing transgenics. Microphotographs of the brains of 30-day-old flies; Bar, 50 µm. **A**) Percentage of the area lost in the regions of the cell body and neuropil: The ratio of lost area was calculated by dividing the sum of the vacuole areas from all brain sections by the total area of the brain from all brain sections. We analyzed 6 brains for each genotype, 16–18 sections per brain. Percentage of the area lost is shown as averages±SEM. p<0.05. **B**) Neurodegeneration in *APP* flies at day 30. Paraffin slices were stained with hematoxylin and eosin and were examined under bright-field illumination using a Leica DM 2500 microscope at a magnification of 120X.

The age-related change in the pattern of n-syb-eGFP fluorescence in *Drosophila* expressing *APP* was accompanied by progressive neurodegeneration. Vacuolization of neuronal tissues is the major hallmark of neurodegeneration in flies [29]. As seen in Fig. 3, all *APP* transgenics demonstrate vacuolar lesions, which are progressive from days 1 to 30. We did not observe vacuolar lesions associated with neurodegeneration in the brains of any transgenic *Drosophila* line on the first day after hatching. On the 15th day, small vacuolar foci appeared in the neuropil and the number of the foci continued to increase with time until day 30. Vacuoles were mostly located in the neuropil while the optic lobes were less affected (Fig. 3). Although the number of vacuoles varied from sample to sample, we did not find significant differences between transgenics that expressed *BACE* and those that lacked *BACE*. All genotypes revealed strong neurodegeneration in comparison with controls (*elav*) [Fig. 3B].

#### Age-dependent learning and memory defects in transgenic flies

All transgenic *Drosophila* lines expressing full length APPs showed reduced learning and memory when tested using an olfactory learning assay [30]. Briefly, 50–100 flies from each line were sequestered in a closed chamber and trained by exposing them sequentially to two odors (octanol or methylcyclohexanol). Flies received electric shock pulses in the presence of the first odor, but not in the presence of the second odor. Trained flies were then tested for conditioned avoidance responses. Immediately after training, learning was measured by allowing flies to choose between the two odors for 120 seconds (Learning Index) or after 1.5 hours (Memory index). The Learning Index and Memory Index for different genotypes are summarized in Tables 1 and 2. In general, a significant decline in learning and memory was observed for all lines expressing full length APPs in neuronal cells. This decline was detected in 1- to 2-day-old flies and became more pronounced as the flies aged.

**Table 1:** Progressive loss of learning ability in *APP*-transgenic flies.

Age of animal Genotype	1–2 days	7–8 days	13–14 days	21–22 days	28–30 days
elav-GAL4^c155^*	61.2±5.2	6.8±3.6	38.6±4.6	34.2±2.8	31.2±6.7
UAS-APP*	58.4±4.7	57.0±6.3	52.7±5.8	44.8±4.4	37.4±7.1
UAS-APP-Sw*	55.4±4.3	42.5±3.0	33.3±3.6	33.7±2.6	35.3±5.3
*elav;APP*	**9.8±4.1**	**9.6±4.3**	**3.6±2.3**	**2.5±1.5**	**2.0±1.9**
*elav;APP/BACE*	**13.6±4.0**	**9.3±2.9**	**6.4±1.8**	**5.3±1.2**	**3.7±2.1**
*elav;APP-Sw*	**22.4±2.4**	**15.3±4.8**	**11.6±4.1**	**4.9±2.8**	**4.0±2.3**
*elav;BACE;APP-Sw*	**28.0±4.2**	**19.3±3.7**	**13.8±3.7**	**8.2±3.0**	**4.2±1.8**

The Learning Index was calculated as described in “Materials and Methods”. All scores are expressed as mean±SEM, (n = 6) where (n) is the number of independent assays. Asterisk (*) indicates control transgenic lines. Statistical analysis was performed using one-way ANOVA and Tukey-Kramer multiple comparison post hoc test. Significance was accepted at p<0.05.

Groups of flies showing statistically significant differences from controls in learning index are shown in bold font.

**Table 2:** Progressive loss of memory in *APP*-transgenic-flies.

Age of animal Genotype	1–2 days	7–8 days	13–14 days	21–22 days	28–30 days
elav-GAL4^c155^*	57.9±3.5	41.5±2.0	30.2±2.3	27.6±1.4	19.7±2.4
UAS-APP*	55.1±5.1	44.8±4.1	36.2±3.6	39.7±3.6	23.4±2.7
UAS-APP-Sw*	53.1±5.7	33.2±3.9	25.0±3.1	28.7±3.4	26.3±3.1
*elav;APP*	**5.0±3.3**	**3.9±2.1**	**3.0±1.6**	**1.2±0.6**	**1.0±0.8**
*elav;APP/BACE*	**13.1±3.3**	**10.4±2.2**	**7.5±1.5**	**4.5±1.7**	**4.9±3.2**
*elav;APP-Sw*	**9.2±1.9**	**7.0±1.5**	**7.7±3.1**	**7.3±2.6**	**5.2±3.3**
*elav;BACE;APP-Sw*	**14.5±4.8**	**13.6±2.5**	**9.1±4.2**	**3.7±1.8**	**4.0±2.5**

The Memory Index was calculated as described in “Materials and Methods”. All scores expressed as mean±SEM, (n = 6) where (n) is the number of independent assays. Asterisk (*) indicates control transgenic lines. Statistical analysis was performed using one-way ANOVA and Tukey-Kramer multiple comparison post hoc test. Significance was accepted at p<0.05. Groups of flies showing statistically significant differences from controls in learning index are shown in bold font.

### II. ApoE-Mimetics and Modulation of AD Neuropathology in Transgenic *Drosophila*

#### In vivo delivery of peptides into brain cells

**Figure 4:**
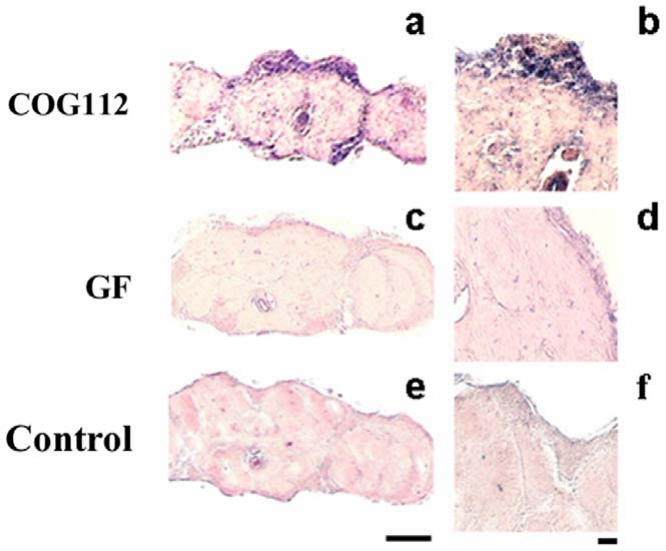
In vivo delivery of peptides into *Drosophila* brain. Immunohistochemistry with antibody to biotin: Left panels - Bar, 50 µm; Right panels-Bar, 10 µm. a,b - Antp-COG133 (COG112) - RQIKIWFQNRRMKWKK LRVRLASHLRKLRKRLL. c,d- CF (fragment 142–153 of human growth factor U2AF)-SQMTRQARRLYV, control peptide, which does not cross BBB [33]; e,f – no peptides added.

ApoE-mimetic peptides are well known to display anti-inflammatory and neuroprotective effects in the central nervous system [21], [22]. Therefore, we tested whether the apoE-mimetic peptides could modify neurodegeneration, amyloidogenesis and cognitive functions in transgenic *Drosophila* models of AD. With the aim of enabling efficient intracellular delivery of apoE-mimetics, we employed protein transduction domain (PTD) technology and evaluated the ability of one of the PTD peptides, penetratin (a 16-amino acid peptide derived from the *Drosophila* Antennapedia homeodomain protein, Antp) to carry a cargo (apoE peptide-COG133) into brain cells. Both penetratin and COG133 crossed the blood brain barrier (BBB) in rodents [21], [31]. However, fusion of Antp to COG133 resulted in significantly enhanced therapeutic effects in animal models [31], [32]. To analyze, whether this fusion peptide is capable of crossing the BBB in *Drosophila*, we injected Antp-COG133 (known as COG112) peripherally into the abdomen, which is filled with the hemolymph that bathes all outer surfaces of the fly brain. To discriminate whether peptide actually crossed the BBB or was simply trapped within the thin layer of perineurial and glial cells, we used microscopy to detect biotin-tagged peptide on whole slices in the parenchymal regions of the brain structures. As seen in Fig. 4, intensity of staining was much higher in the outer cellular cortex layer containing neuronal and glial cells when compared with neuropil areas. However, at this level of resolution, it is difficult to conclude whether the biotin-tagged peptide penetrated inside specific brain cells. Experiments without added peptides or with injected control peptide CF, which does not cross BBB [33], demonstrated only very weak staining.

#### Effect of peptides on neurodegeneration

**Figure 5:**
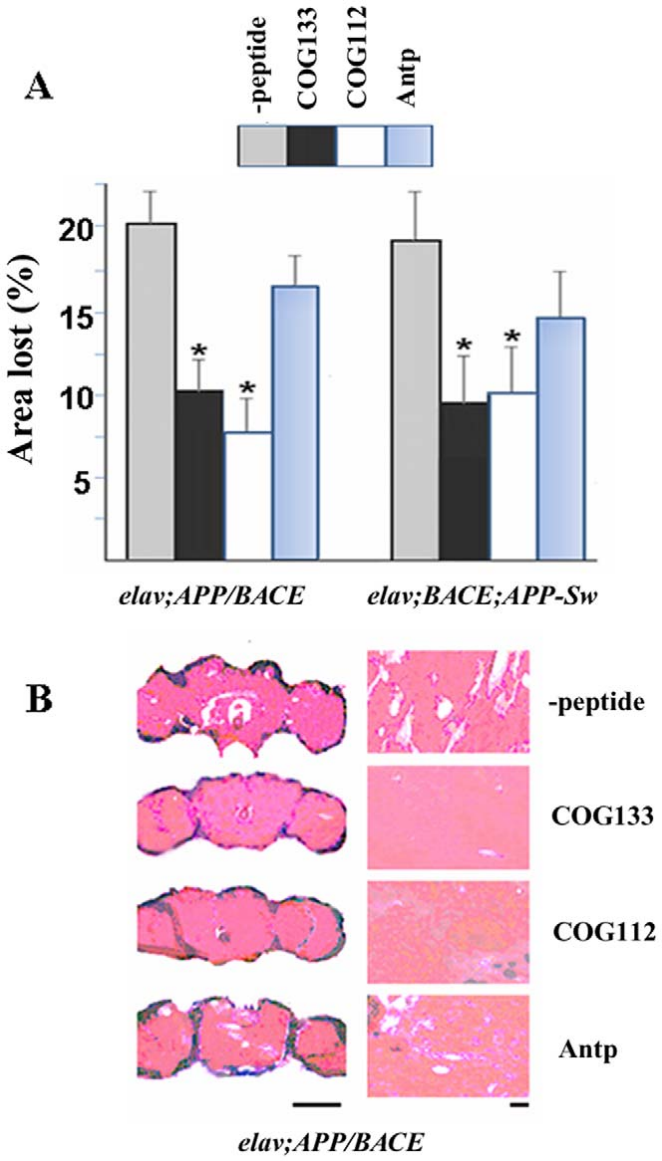
ApoE-mimetics inhibit neurodegeneration in *Drosophila* brain. **A**) Effect of peptides on percentage of the area lost in the regions of the cell body and neuropil: The ratio of lost area was calculated by dividing the sum of the vacuole areas from all brain sections by the total area of the brain from all brain sections. Percentage of the area lost is shown as averages±SEM. p<0.05 Asterisks indicate significant differences from control. **B**) Effect of peptides on neurodegeneration in *APP* transgenic flies. Paraffin slices were stained with hematoxylin and eosin and were examined under bright-field illumination using a Leica DM 2500 microscope at a magnification of 120X. Left panel- Bar, 50 µm; Right panels - Bar, 10 µm.

The development of vacuolar defects was almost entirely ameliorated by injections of COG112 or COG133 in *elav;APP/BACE* and *elav;BACE;APP-Sw* transgenic lines [Fig. 5]. Amounts of different sized vacuoles in the neuropil of the central brain and optical lobes were drastically decreased after seven injections of apoE peptides. The protective effect was significant even on day 15–17 day following the fourth injection of peptides. In control experiments, injections of penetratin (Antp) in the same doses did not block neurodegeneration. Six brains for each genotype and 16–18 sections per brain were analyzed. Significant differences from controls (p<0.05) were observed for COG112 and COG133 for both tested genotypes (*elav;APP/BACE* and *elav;BACE;APP-Sw*).

#### Effect of peptides on learning and memory

To clarify whether apoE-mimetics could inhibit vacuolarization associated with neurodegeneration and help to restore cognitive functions, we analyzed the effects of COG112 or COG133 on learning and memory in two *APP* expressing lines: *elav;APP/BACE* and *elav;BACE/APP-Sw*. These lines showed the strong vacuolarization associated with neurodegeneration and a reduction of synaptic protein levels in mushroom bodies, centers for olfactory learning and memory. After two injections over the first 7 to 8-days of age, COG112 or COG133 treatments did not show any effects on learning in flies with the *elav;APP/BACE* genotype. With 3 additional treatments of COG112 or COG133, however, significant positive effects on restoring learning and memory abilities were observed for COG112 and for COG133 by days 21–22 of age. For the *elav;BACE; APP-Sw* genotype that displayed severe vacuolarization and decreased levels of synaptic proteins, statistically significant restoration of memory was only detected with COG133 in 7-8-day-old flies. These effects of apoE-peptides on learning and memory are summarized in Table 3.

**Table 3:** Effect of COG133 and COG112 on learning and memory in *APP*-nransgenic flies.

Genotype	Peptide injection	Learning Index (%)		Memory Index (%)	
		7–8 days	21–22 days	7–8 days	21–22 days
*elav;APP/BACE*	**− petide**	9.3±2.9	5.3±1.2	10.4±2.2	4.7±2.0
	**+Cog133**	11.6±1.3	**9.8±2.5**	**18.0±2.6**	6.2±1.4
	**+Cog112**	10.0±2.7	**8.6±1.2**	**17.6±1.4**	**12.2**±2.3
	**+Antp**	6.0±1.1	6.2±1.3	6.1±2.7	3.6±1.5
*elav;BACE;APPSw*	**− petide**	19.1±4.1	8.2±3.0	13.6±2.5	3.7±1.8
	**+Cog133**	25.4±1.9	9.3±3.5	**19.6±1.2**	3.7±1.4
	**+Cog112**	21.7±3.1	11.3±1.4	15.3±1.7	5.2±2.7
	**+Antp**	28.7±6.5	10.7±1.7	10.4±2.1	4.0±1.1

The Learning Index and Memory Index were calculated as described in “Materials and Methods”. All scores expressed as mean±SEM, (n = 6) where (n) is a number of independent assays. Statistical analysis was performed using one-way ANOVA and Tukey-Kramer multiple comparison post hoc test. Significance was accepted at p<0.05. Groups of flies showing statistically significant recovery of learning and memory in comparison with control (Antp) are shown in bold font. 0.1 µl injections of 11.74 µM COG133, 11.47 µM COG112, and 11.71 µM Antp into the abdomen were performed every 4 days starting from day 2.

#### Effect of peptides on Aβ deposition

**Figure 6:**
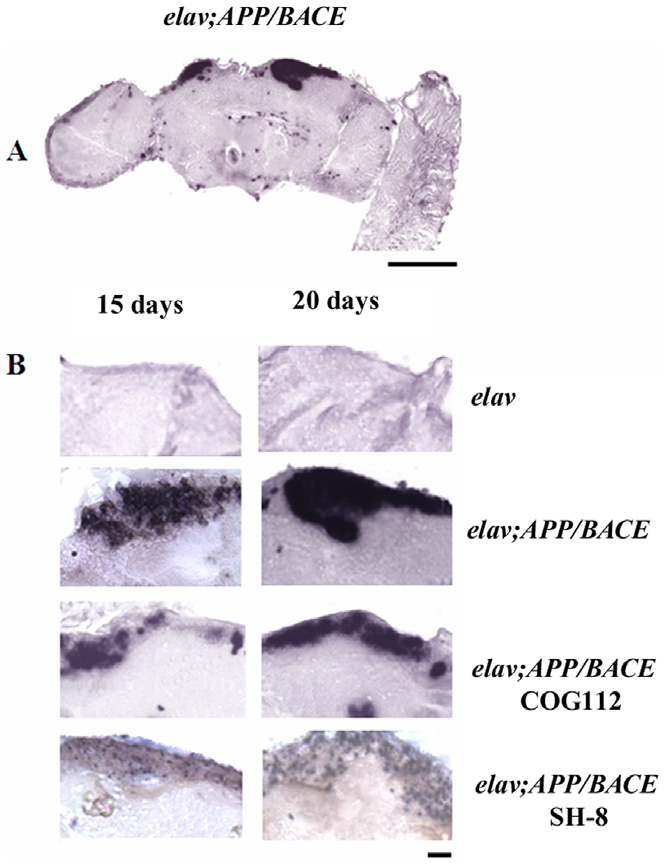
Effect of peptides on Aβ accumulation in *Drosophila* brain. Immunohistochemistry with 4G8 antibody: **A**) Distribution of Aβ deposits in *Drosophila* brain, Bar, 50 µm. **B**) Effect of peptides on Aβ accumulation in *Drosophila* brain, Bar, 10 µm.

Although our results show that apoE-mimetics appear to restore cognitive functions in *APP* transgenic flies, we did not know if this effect resulted from interfering with Aβ pathology. To address this point, we investigated the effects of COG112 on Aβ accumulation in *Drosophila* brain. We analyzed the effect of oral administration of COG112 (aka. Antp-COG133) to an *APP* transgenic line with the *elav;APP/BACE* genotype. This line has been characterized by a high level of Aβ deposits accompanied by strong age-related neurodegeneration and a significant reduction in the levels of synaptic proteins. We chose to test COG112 because previous experiments showed that COG112 was more efficiently delivered into cells and it was more stable in experiments *in vivo* [32]. Flies were cultured on standard fly food containing tested peptides, which were added every day at concentrations: 11.47 µM COG112, 11.71 µM Antp, or 3.72 µM Antp-SH8. SH8-represented a previously tested inhibitor of Aβ aggregation [34]. As seen in Fig. 6A, significant Aβ deposition in the *Drosophila* brain was detected in the cortical layer containing neurons. Much less Aβ deposition was detected in the neuropil. Oral administration of Antp-SH8 remarkably decreased the size and density of Aβ-positive deposits. Antp alone did not appear to change Aβ accumulation in the brain (Fig. 6B). Several brain samples showed decreased size of Aβ deposits after COG112 administration, but the effect of this peptide is difficult to evaluate due to significant variations in detectable Aβ deposits. Most likely these results suggest a slight effect of this peptide on Aβ levels, which will require more refined methodologies to precisely measure.

Functional integrity of peptides during oral administration was tested by comparison of their effects on neurodegeneration with effects of the same peptides after abdominal injections. COG112, COG133 and Antp-SH8 all inhibited neurodegeneration to an equal degree that was independent of the method of peptide delivery, while Antp alone had no effect (Table 4).

**Table 4:** Comparison of prevention of neurodegeneration in the brain of *APP*-transgenic flies by apoE-mimetics after injection and feeding.

Genotype	Control (-peptide) Degenerated area	Injection Degenerated area	Feeding Degenerated area
**Cog133**			
**elav; APP/BACE**	20,0±0,8	**11,7±1,2**	**9,5±0,6**
**elav; BACE; APP-Sw**	18,3±2,0	**8,7±0,6**	**8,6±1,3**
**Cog112**			
**elav;APP/BACE**	20,0±0,8	**7,6±0,8**	**11,4±1,1**
**elav; BACE; APP-Sw**	18,3±2,0	**10,8±0,6**	**9,9±0,9**
**SH8**			
**elav;APP/BACE**	20,0±0,8	**ND**	**13,4±1,3**
**elav; BACE; APP-Sw**	18,3±2,0	**ND**	**11,6±0,7**
**Ant**			
**elav;APP/BACE**	20,0±0.8	16,6±0,9	18,9±1,1
**elav; BACE; APP-Sw**	18,3±2,0	14,8±1,3	13,6±1,0

The degenerated area was calculated as described in Materials and Methods at day 30. Six brains were analyzed for each genotype. Statistical analysis was performed using one-way ANOVA and Tukey-Kramer multiple comparison post hoc test. Significance was accepted at p<0.05. Results showing statistically significant decrease of neurodegeneration in comparison with control are shown in bold font. Flies were cultured on standard fly food containing tested peptides, which were added every day at concentrations of: 11.47 µM COG1112, 11.71 µM Antp, and 3.72 µM Antp-SH8.

0.1 µl injections of 11.74 µM COG133, 11.47 µM COG112, and 11.71 µM Antp into the abdomen where performed every 4 days starting from day 2. All scores expressed as mean±SEM (n = 6).

## Discussion

A large number of studies implicated defective processing of APP and formation of neurotoxic Aβ oligomers as a main cause of synaptic dysfunction in AD [4], [35]. However, the idea that only enhanced production of Aβ oligomers may lead to neurodegeneration and synaptic dysfunction has been challenged by recent results, suggesting that impaired functions of PS1, PS2 and/or APP may contribute to AD-like synaptic pathology in a fashion that is independent from Aβ generation. Indeed, conditional *PS1* and *PS2* knockout mice have been characterized by classical hallmarks of AD neuropathology including tau hyperphosphorylation, progressive neurodegeneration, impairment of synaptic plasticity and memory loss, all in the absence of Aβ [36], [37]. In parallel, deficits in synaptic plasticity and cognitive functions were detected in *APP* knockout or *APP* knockdown animal models [38], [39]. These results suggest that the presence or absence of APP, independent of Aβ, may lead to some form of synaptic pathology and dysfunction.

Several studies using transgenic Drosophila were directed to discriminate effects of exogenous APP and Aβ. Transgenic flies directly expressing Aβ-42 in the brain demonstrated diffuse amyloid deposits and age-dependent learning defects in olfactory learning [40]. In other work, flies expressing wild-type Aβ-42 and Arctic mutant Aβ-42 (Glu22Gly) showed a decline in climbing behavior, increased intracellular Aβ accumulation and diffuse plaques prior to signs of neurodegeneration [41]. Recent findings demonstrated that expression of the Arctic mutant significantly enhanced formation of Aβ oligomers and Aβ deposits, together with a decline of locomotor functions when compared with Aβ-art (artificial mutation L17P) [42]. Surprisingly, different Aβ-42 aggregates have distinctive roles in modulation of synaptic functions. While exogenously prepared small Aβ-42 oligomers or Aβ oligomers secreted from neurons lead to a reduction of neurotransmitter release; larger-sized aggregates, possibly fibrils secreted by muscle cells, enhanced neurotransmitter release and synaptic transmission (43).

Because BACE activity is not present in *Drosophila*, overexpression of human *APP* does not lead to secretion of Aβ leading to the interpretation that all phenotypic effects in these transgenic flies should be attributed to the presence of human APP. Although APPL processing by endogenous BACE-like enzymes in the fly could result in accumulation of unusual APPL fragments [44], transgenics with *elav; APP* genotypes express human APP at levels much higher than the endogenous APPL [20], [26]. When human BACE and APP were expressed in combination in fly eyes, diffuse amyloid plaques and age-dependent neurodegeneration of photoreceptor cells were observed [20]. Surprisingly, neurodegeneration was even more pronounced in *APP* transgenic flies than in *APP/BACE* double transgenic flies.

Our data confirm these results where APP alone, or APP plus Aβ, may result in different degrees of impairment. We did not find differences in age-dependent neurodegeneration in transgenic flies expressing full size APP with BACE or without BACE (Fig. 2). However, transgenics expressing APP and BACE had lower levels of the presynaptic protein GFP-n-synaptobrevin than transgenics expressing APP alone (Fig. 2B). These findings raise the question whether the decline of synaptic proteins levels and/or neurodegeneration are caused by different mechanisms. Alternatively, we suggest that Aβ represents just a part of a larger pathological process and independently contributes to different neuropathological abnormalities caused by APP overexpression. Although the effect of Aβ on reduction of n-syb-eGFP level was readily observable, we conclude that overexpression of full length APP may be sufficient for abnormal synaptogenesis in the *Drosophila* brain. We previously obtained similar data for another presynaptic protein: GFP-n-synaptotagmin [45]. Interestingly, only full length APP overexpression caused a disruption of axonal transport in *Drosophila* [46].

Most of the described *APP* transgenic models exhibit learning and memory deficits that reflect a clinical syndrome associated with APP pathogenesis [47], [48]. In our experiments, we detected reduced learning and memory in 2 day-old flies, while significant neurodegeneration and Aβ accumulations were observed in 10–15 day-old flies. (Figs. 3 and 6). While the molecular basis for this finding is not clear, early decline of cognitive functions supports a primary role for the synaptic dysfunction in these transgenic models.

A reasonable approach to understand these results is based on testing compounds that would prevent (or reverse) impairment of cognitive functions and simultaneously block neurodegeneration, and/or oligomerization of Aβ. We tested the effects of injected apoE-mimetic peptides with known neuroprotective activity in different animal models of neurological diseases [22]. A major obstacle in this approach is the limited penetration of peptides into the brain. Therefore, we fused the COG133 apoE-peptide with penetratin to generate COG112 [22], which was previously successfully tested for the transport of cargo across the BBB in *Drosophila* and rodents [31], [32], [49], [50]. We tested these peptides in *elav; APP/BACE* and *elav;BACE/APP-Sw* lines that display age-progressive neurodegeneration and strong declines in cognitive functions. Both COG112 and COG133 blocked neurodegeneration and restored learning in the flies after abdominal injection or oral administration after two injections before 7–8 days of age. However, restoration of memory function was detected only in the *elav;APP/BACE* line by 20–22 days of age and after 5 injections of COG compounds.

To further understand the mechanism of neuroprotection by apoE-peptides, we analyzed their effect on Aβ deposition in transgenic flies. Expression of APP in the presence of BACE caused significant accumulation of Aβ in the cortical layer containing neuronal cells, glial cells and small Aβ deposits in the neuropil of 10–15 day old flies (Fig. 6A). SH-8 significantly decreased the size and density of Aβ deposits (Fig. 6B) supporting our previous observation that SH-8 inhibits amyloid growth [34]. Although some brain samples show decreased size of Aβ deposits after COG112 administration, the influence of this peptide on Aβ metabolism is difficult to interpret due to significant variations in the sizes of detectable Aβ deposits. Taking into consideration that we found COG112 to partially suppress elevation of Aβ 1–42 levels in mice after head injury [23], these results may only suggest a slight effect of this peptide on Aβ metabolism. In summary, neuroprotective effects of apoE-mimetics in Drosophila probably have multiple features that are not solely attributable to Aβ-mediated neurotoxicity.

Experiments describing restoration of cognitive functions in transgenic animals demonstrate that protective effects may be achieved at different levels. Administration of an ubiquitin C-terminal hydrolase L1 (Uch-L1)—an enzyme associated with the breakdown of excess or abnormal proteins, has a protective effect on memory loss in a mouse model of AD (APP Sw-PS1M146V) [51]. These authors showed that effect of the Uch-L1 is associated with restoration of phosphorylation of the cAMP response element binding protein (CREB). An increase in phospho-CREB levels may stabilize synaptic connections and may contribute to long-term memory formation.

Administration of the M1 muscarinic agonist, AF267B, can also rescue selected deficits in Alzheimer's models. The effect of AF267B on cognition predicted changes in neuropathology that were observed to be reductions in Aβ and tau pathologies in the hippocampus and cortex. The mechanism underlying this effect on the Aβ pathology was caused by the selective activation of ADAM17 by AF267B treatment, thereby shifting APP processing toward the non-amyloidogenic alpha-secretase pathway. Reductions in tau pathology appear to be mediated by decreased GSK3β activity [52]. Finally, the ionophore 8-hydroxy-quinoline, which targets metal-induced aggregation of Aβ, significiantly decreased soluble interstitial brain Aβ within hours and improved cognitive performance in *APP* transgenic mice [53].

The precise natures of neuroprotective functions of apoE mimetics are not yet clear and several mechanisms have been suggested. A recent report showed that the activity of apoE (133–149) mimetic peptide depends on the low-density lipoprotein receptor-related protein (LRP). Binding LRP by apoE(133–149) results in inhibition of the N-methyl-D-aspartate (NMDA) receptor (NMDAR). Electrophysiology experiments demonstrated that the inhibitory potency of apoE(133–149) was threefold greater for NMDAR transfected wild-type Chinese hamster ovary (CHO) cells containing LRP compared with NMDAR-transfected CHO cells deficient in LRP [54]. Another report shows that COG112 inhibits the inflammatory response to *Citrobacter rodentium* in colonic epithelial cells by preventing NFκB activation [55]. This is an important observation to help explain the effects of COG112 on neurodegeneration in *Drosophila* because the Toll→NFκB signaling pathway mediates some neuropathological effects in *Drosophila* [56].

In conclusion, our study suggests that multiple cellular mechanisms are involved in restoring cognitive functions in APP flies. This study with apoE-mimetics indicates that new approaches to discover these mechanisms may lead to the development of new therapeutics for the successful treatment of AD.

## Materials and Methods

### Lines of Drosophila and Maintenance Conditions

We used the following transgenic lines of *D. melanogaster*: *UAS–APP* (carrying the human *APP695*), *UAS–APP–Swedish* (carrying the human *APP69 5* with the 670K3N, 671M3L mutations, *UASAPP* Δ*CT, UAS-APP*Δ*NT* (truncated forms of *APP*695), *elav–GAL4c155*, *UAS–n-syb–eGFP* (eGFPn- synaptobrevin), *UAS-BACE* (carrying the human *BACE)*. Expression of h*APP* and its truncated forms in neural cells of *Drosophila* was driven by the tissue-specific transcription driver *elav–GAL4c155*. The UAS-BACE was kindly provided by R. Reifegerste, the other stocks were obtained from the collection of the *Drosophila* Bloomington Stock Center. During the study, the flies were kept on the standard yeast medium at a temperature of 29°C and a photoperiod of 12 h.

Synaptobrevin expression was analyzed in the offspring of crosses between *elav–GAL4c155*,*UAS–n-syb-eGFP* (aka. *syb*) females and males carrying insertions of *APP* (*APP*–*Swedish*), insertion of *BACE* and genes of truncated *APP* forms. Transgenic lines were designated in text as following *elav-GAL4c155;UAS-APP* (aka. *elav;APP*), *elav-GAL4c155;UAS- APP/UAS-BACE* ***(***aka. *APP/BACE*), *elav GAL4c155;UAS-APP-Swedish* (aka. *elav;APP-Sw*), *elav-GAL4c155; UAS-BACE; UAS- APP-Swedish* (aka. *BACE/APP-Sw*).

### Western Blotting

Fly heads were homogenized in lysis solution containing 1x PBS, 5 mM EDTA, 0.5% Triton X-100, and a complete protease-inhibitor mix (Roche Applied Science, Mannheim, Germany). Equal amounts of protein were separated by 10% SDS-PAGE, transferred to Immobilon membranes (Millipore, Bedford, MA), blocked in 5% low-fat milk for 2 hr at room temperature, and incubated with the monoclonal antibody 22C11 (APP N terminal-specific; Chemicon, Temecula, CA). Bound antibodies were detected with goat anti-mouse peroxidase-conjugated secondary antibody (Sigma, St. Louis, MO). For immunoprecipitation, frozen pellets of fly heads were homogenized as described above, and lysates were treated with protease and phosphatase inhibitors (Sigma, St. Louis, MO). After pelleting insoluble material, the lysate was incubated with anti-Aβ monoclonal 4G8 (Signet Laboratories, Dedham, MA) antibody (0.3 µg/ml of lysate) on ice for 90 min, and then the antibody/antigen complexes were recovered by incubation with protein G-Sepharose beads. After pelleting and washing of the beads, recovered proteins were fractionated by SDS/PAGE on 4–20% polyacrylamide Novex gels (Wadsworth, OH ) and transferred to Immobilon membranes (Millipore, Bedford, MA), blocked in 5% low-fat milk for 2 hr. Bound antibodies were detected with goat anti-mouse peroxidase-conjugated secondary antibody (Sigma, St. Louis, MO). All blots were scanned and quantification of the relative intensity of each band was evaluated by using the NIH Image J program (http://rsb.info.nih.gov/ij/index.html). This method is outlined in the Image J documentation: “Gel Analysis.”

### Preparation of Specimens for Confocal Microscopy

The flies were immersed in fixing solution (3.38 ml of 37% formaldehyde (Merck), 0.5 ml of 1 M Na2PO4 (pH 6.8), 5 ml of octane, and 1.12 ml of water) for 20 min. Fixed heads were separated from the body in a phosphate buffer solution and put into a second fixing solution (0.43 ml of 37% formaldehyde (Merck), 0.4 ml of 1 M Na2PO4 (pH 6.8), and 3.17 ml of water) for 90 min at 4°C. After fixation, the brains were isolated in a phosphate buffer solution and placed on glass plates with hollows containing a 1∶1 mixture of phosphate buffer solution and glycerin.

### Confocal Microscopy and Estimation of Fluorescence of GFP–n-synaptobrevin

An LSM5 Pascal confocal microscope with a built-in 35-mW argon laser was used for confocal microscopy. All samples were scanned with the same scanning settings and the fluorescent signal was visualized at λ 488 nm. The slices for scanning were 2 µm in thickness. The resultant images and series of images were analyzed using the LSM5 Image Browser software. The fluorescence intensity was evaluated in microphotographs of confocal slices using the Image J software (version 1.38a for Windows). (http://rsb.info.nih.gov/ij/index.html). The methods of quantification are outlined in the Image J documentation: “Image → Staks” and “Analyze →Measure”. Images of 2 µm optical sections were converted to stacks, and regions containing mushroom bodies and antennal lobes were demarcated using the “Freehand tool”. Pixel intensity in the selected areas was estimated using Image J software. For calculation of relative intensities the pixel intensity was normalized to the fluorescent signal of *elav* genotype. Four brains were analyzed for each genotype. Related intensities are shown in means±SEM. Statistical analysis was performed using one-way ANOVA and Tukey-Kramer multiple comparison post hoc test. Significance was accepted at p<0.05

### Peptides, Abdominal Injections, Oral Administrations

Peptides used for studies included Penetratin (Antp 43–58) -RQIKIWFQNRRMKWKK; CF (fragment 142–153 of human growth factor U2AF)-SQMTRQARRLYV, Antp-COG133 (COG112)-RQIKIWFQNRRMKWKKCLRVRLASHLRKLRKRLL, COG133-LRVRLASHLRKLRKRLL, SH8 – RHVLPKVQA. Antp-SH8: RQIKIWFQNRRMKWKK-RHVLPKVQA. An N-terminal tag of biotin-γAbu (gamma-aminobutyric acid) was added to penetratin (Antp), COG133, Antp-COG133 (COG112), CF (U2AF, 142–153). Untagged peptides COG133, Antp-COG133 and SH8 were synthesized by the solid phase technique on an ABI Model 430A synthesizer. Peptides were then deprotected by treatment with anhydrous hydrogen fluoride in presence of m-cresol and purified by preparative reverse-phase chromatography. Purified peptides were characterized by analytical RP-HPLC using a DeltaPak C-18 column with a 20–50% gradient of acetonitrile in 0.1% trifluoroacetic acid at 1 ml/min. Amino acid analysis was performed following complete hydrolysis in 6N HCl for 24 hr at 110°C (LKB 4151 Alpha Plus amino acid analyzer, Sweden) and mass-spectrum analysis was performed on a Voyager-DE BioSpectrometry Workstation (PerSepetive Biosystems, USA). Purity of all peptides used in this study was >95%.

Abdominal injections were performed as we described in [56]. After dissolving peptides in Ringer' solution (7.5 g/liter NaCl/0.35 g/liter KCl/0.2g/liter CaCL2; pH 7.6–7.8), they were injected in a volume of 0.2 µl per fly into the abdomen using glass pipettes (20–40 µm tip diameter) coupled to an injector and micromanipulator. Peptides were injected on days 2, 5, 9, 13, 17, 21, 25 and 29 post-eclosion. For oral administration, tested peptides were added every day to standard fly food at final concentrations of 11.47 µM COG133 and COG 112, 11.71 µM Antp, 3.72 µM Antp-SH8.

### Immunohistochemistry

For immunostaining flies were fixed in freshly prepared Carnoy's fixative for 24 hours. Next, flies were dehydrated sequentially in 30, 50, 75, and 100% ethanol and embedded in paraffin. 6 µm thick sections were blocked for 1 hr in 0.5% nonfat dry milk, 0.1% Tween-20 in PBS, washed two times with 0.1% Tween-20 in PBS and two times with PBS. Slides were then incubated with anti-Aβ monoclonal 4G8 (Signet Laboratories, Dedham, MA) (1∶500) (or antibody to biotin- Sigma, St.Louis, MO) for 2 hours, washed four times with PBS and incubated with goat anti-mouse phosphatase-conjugated secondary antibody (Sigma, St. Louis, MO) for 2 hours. Slides were washed with PBS. Nitroblue tetrazolium chloride and 5-bromo-4-chloro-3-indolyl phosphate substrate (1-STEP NBT/BCIP, Pierce) was added to slides until desired stain intensity develops. Finally, slides were rinsed with water.

### Neurodegeneration Assay

The fly heads were fixed in freshly made Carnoy's fixative for 24 h at a temperature of 4°C, embedded in paraffin, and 5 µm paraffin slices were prepared as described [25]. Paraffin slices were stained with hematoxylin and eosin (Bio Optica, Italy). Next, slices were examined under bright-field illumination using a Leica DM 2500 microscope at magnification of 120X. To quantify neurodegeneration in the cell body and neuropil, images of the 5 µm brain sections stained with hematoxylin and eosin and were captured using bright-field microscopy. The areas of the vacuoles in the cell body and neuropil regions on each brain section were measured using NIH Image J software (http://rsb.info.nih.gov/ij/index.html). The method of quantification is outlined in the Image J documentation: “Particle Analysis.” The ratio of lost area was calculated by dividing the sum of the vacuole areas from all sections by the total area of the brain from all sections. Percentage of the area lost is shown as average±SEM. We analyzed 6 brains for each genotype, 16–18 sections per brain. Statistical analysis was performed using one-way ANOVA and Tukey-Kramer multiple comparison post hoc test. Significance was accepted at p<0.05

### Olfactory Associative Learning

Olfactory associative learning was performed as described on 50 to 100 flies per group [30]. Flies were trained by exposure to electroshock paired with one odor: octanol or methylcyclohexanol, for 60 s and subsequent exposure to the other odor without electroshock for 60 s. Immediately after training, learning is measured by allowing flies to choose between the two odors for 120 s (Learning Index) or after 1.5 hour (Memory index). Performing index (P.I.) (Learning Index or Memory Index) was calculated as the number of flies that responded correctly (to avoid the shock-paired octanol) minus the number of flies that responded incorrectly (to avoid the control odor methylcyclohexanol) divided by the total number of flies. To eliminate naive odor bias, each trial was composed of two half-trials, where one group was trained to associate octanol with shock and the other to associate methylcyclohexanol with shock, and the complete P.I. was the average of these two half-trial P.I.s. Statistical analysis was performed using one-way ANOVA and Tukey-Kramer multiple comparison post hoc test. Significance was accepted at p<0.05.

### Statistics

Statistical analyses were performed using the *KyPlot* software (KyensLab Inc). One-way ANOVA was followed by planned multiple comparisons between relevant groups with Tukey-Kramer test.
